# Corrosion-Resistant and Conductive Coatings on 316L Stainless Steel Bipolar Plates Fabricated by Hot Rolling

**DOI:** 10.3390/ma18081831

**Published:** 2025-04-16

**Authors:** Xiaojun Zhao, Zihao Wang, Lairong Xiao, Yitao Zha, Guanzhi Deng, Shaohao Li, Zhenyang Cai, Sainan Liu

**Affiliations:** 1 School of Materials Science and Engineering, Central South University, Changsha 410083, China; zhaoxj@csu.edu.cn (X.Z.); cn_wangzihao@163.com (Z.W.); xiaolr@csu.edu.cn (L.X.); 233111018@csu.edu.cn (Y.Z.); 223111043@csu.edu.cn (G.D.); lishaohao2022@163.com (S.L.); 2 State Key Laboratory of Powder Metallurgy, Ministry of Education, Central South University, Changsha 410083, China; 3 State Key Laboratory for Light Weight and High Strength Structural Materials, Central South University, Changsha 410083, China; 4 School of Minerals Processing and Bioengineering, Central South University, Changsha 410083, China

**Keywords:** bipolar plates, coating, corrosion resistance, conductivity, hot rolling

## Abstract

The insufficient corrosion resistance and high interfacial contact resistance (ICR) of 316L stainless steel (316L SS) severely limit its application as bipolar plates (BPs) in proton exchange membrane fuel cells (PEMFCs). In this study, a graphite/carbon black/PVDF composite coating was first developed by hot rolling on the surface of 316L SS to enhance both corrosion resistance and conductivity. By incorporating 5 wt% polyaniline (PANI) as a corrosion inhibitor, the optimized RP5 coating exhibited further improvements in corrosion resistance. The potentiodynamic polarization tests revealed that the RP5 coating achieved a corrosion current density of 0.977 μA·cm^−2^, representing a two-orders of magnitude reduction compared to bare 316L SS (34.1 μA·cm^−2^). The coating also exhibits a satisfactory interfacial contact resistance (ICR) of 8.20 mΩ·cm^2^ at 1.5 MPa, meeting the U.S. Department of Energy (DOE) 2025 targets (<10 mΩ·cm^2^). Additionally, the RP5 coating exhibited superior hydrophobicity with a water contact angle of 96.5°, which is advantageous for water management within PEMFCs. The results confirm that the RP5 coating achieves an optimal balance between high conductivity, excellent corrosion resistance, and improved hydrophobicity, making it a promising solution for advancing PEMFC bipolar plates’ performance.

## 1. Introduction

Proton exchange membrane fuel cells (PEMFCs), as devices that directly convert hydrogen energy into electrical or other forms of energy, have advantages such as low start-up temperature and minimal pollution [1,2]. As a core component of PEMFC, the bipolar plates constitute 40% of the total cost and 50% of the total volume of the PEMFC stack [2]. The main functions of the bipolar plates include separating and conducting reaction gases, conducting electric current, and supporting the membrane electrode [3]. Therefore, to fulfill their functional requirements, bipolar plates necessitate high electrical conductivity, enhanced anti-corrosive properties, suppressed gas diffusion characteristics, and exceptional mechanical robustness [4].

Nowadays, bipolar plates are classified according to material type into the following three main categories: graphite, metal, and composite bipolar plates [5,6,7]. However, both graphite and composite bipolar plates continue to encounter challenges, including low conductivity, limited flexural strength, and excessive thickness [8,9]. Consequently, research has shifted toward ultra-thin metal bipolar plates, such as stainless steel [10], titanium-based alloys [11], and aluminum-based alloys [12]. Among these, 316L stainless steel (316L SS) has emerged as a leading candidate due to its cost-effectiveness and excellent mechanical properties [13].

Although 316L SS is corrosion-resistant, it remains susceptible to corrosion in the highly acidic and basic environments of PEMFCs. During the corrosion process, the released metal ions such as Fe^3+^ and Cr^3+^ would contaminate the catalyst layer, ultimately leading to the failure of the fuel cell stack [14,15]. Therefore, surface treatments are essential for 316L SS. Coatings such as carbon-based materials [16], inert metal coating (Au) [17], or metal compounds (CrN) [18] are applied to the 316L surface to enhance the corrosion resistance. Haghighat Ghahfarokhi et al. [19] applied a-C coatings to 316L bipolar plates by closed field unbalanced magnetron sputtering. It was observed that a bias of 120 V produced the densest cross-structure and minimized the corrosion current density. Wang et al. [20] deposited molybdenum carbide (MoC) coatings with varying thicknesses onto 316L stainless steel via magnetron sputtering, achieving a remarkably low corrosion current density of 0.23 μA·cm^−2^; however, these coatings are typically prepared using physical vapor deposition (PVD). Although these techniques are widely used, they require sophisticated and costly equipment, resulting in high capital investment. To facilitate the commercialization of PEMFC, the low-cost polymer-based composite coatings remain a critical direction for future research [21].

Show et al. [22] manufactured a carbon nanotube (CNT) and polytetrafluoroethylene (PTFE) composite coating. The unique composition of this coating enabled it to possess excellent corrosion resistance, which is vital for maintaining the integrity of PEMFC components in harsh operating environments. Nevertheless, in their study, the long-term corrosion test was not provided. Sharma et al. [23] investigated PANI-TiN composite coatings on SS304 substrates. The composite coatings exhibited an ICR of 32.6 mΩ·cm^2^, compared to 367.5 mΩ·cm^2^ for pristine PANI, which is still far from the Department of Energy (DOE) target. Husby et al. [24] investigated the corrosion resistance of 316L stainless steel (316L SS) bipolar plates by fabricating a polymer composite coating. Electrochemical tests demonstrated the coating’s significant protective effect on the 316L substrate. However, this protective effect diminished with increasing corrosion potential, with coating failure initiating at 1.0 V. In general, polymer-based composite coatings manufactured by traditional methods, such as blade coating or electrochemical deposition, often face challenges in balancing the electrical conductivity and corrosion resistance, primarily due to their loose microstructure and the ratio of conductive fillers to polymer resin matrices [25].

In this study, a graphite/carbon black/PVDF composite coating was first developed on the surface of 316L SS through the hot rolling process, which can significantly enhance the corrosion resistance and conductivity of coating. Moreover, as a continuous production process, the hot rolling process has the advantages of high production efficiency and low equipment requirements, which are conducive to reducing the production cost of bipolar plates. Additionally, the corrosion resistance of the coating was further improved by adding polyaniline (PANI) as a corrosion inhibitor.

## 2. Materials and Methods

### 2.1. Pre-Treatment of 316LSS

**Figure 1:**
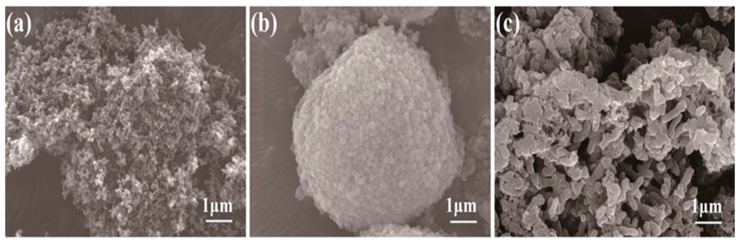
SEM images of the particles: (**a**) carbon black; (**b**) PVDF; and (**c**) PANI.

The 316L SS was used as the substrate material. The surface of the stainless steel was polished using 240-grit sandpaper, followed by alkaline cleaning, degreasing, and ultrasonic cleaning to ensure a clean surface. Finally, the 316L stainless steel was cut into 15 mm × 100 mm × 0.1 mm pieces to be applied in subsequent experiments. SEM images of the particles were shown in Figure 1.

### 2.2. Coating Preparation

**Figure 2:**
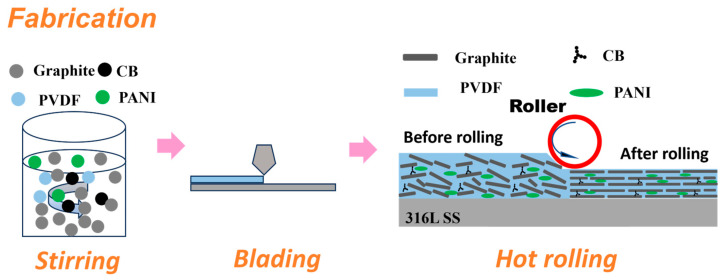
The preparation process of composite coating by means of blade and hot rolling.

A semi-dry method was used to prepare the slurry. A predetermined ratio of high-purity graphite (300 mesh) and conductive carbon black powder were mixed in a ball mill for 1 h. Polyaniline, polyvinylidene fluoride (PVDF), and N-methylpyrrolidone were subsequently added to form a uniform slurry. A pre-coating is prepared on the surface of 316L SS by blade coating, and then the coating is further compacted through a hot rolling process to improve its electrical conductivity and corrosion resistance. The detailed process steps are shown in Figure 2.

The contrast sample P0 coating (0 wt% PANI) was prepared by blade. The RP0 (0 wt% PANI), RP5 (5 wt% PANI), and RP15 (15 wt% PANI) coatings were prepared by blade and hot rolling. The sample identification, composition, and preparation processes are detailed in Table 1, with PANI representing the additional amount added.

### 2.3. Analyses

#### 2.3.1. Microstructure Characterization

The micro-morphology of the composite coatings was observed using scanning electron microscopy (SEM, Tescan Mira4, Brno, Czech Republic). The distribution of the binder and polyaniline within the composite coating was analyzed using energy dispersive spectroscopy (EDS, Oxford Xplore30, Oxford, UK). The surface roughness of the samples was measured with an optical profilometer (Wyko NT9100, Billerica, MA, USA), which uses interferometry-based optical microscopy to capture 2D images of the coated substrates, allowing for an average surface roughness (Ra) calculation.

#### 2.3.2. Electrochemical Testing

To evaluate the electrochemical performance of the composite bipolar plates, a standardized three-electrode electrochemical cell was employed. The working electrode comprised a precisely machined sample with an exposed geometric surface area of 1 cm^2^, and a platinum sheet functions as the counter electrode, while a saturated calomel electrode is used as the reference electrode. The corrosive operational environment of PEMFC was simulated using a 0.5 M sulfuric acid (H_2_SO_4_) electrolyte. The corrosion resistance of the sample was characterized using potentiodynamic polarization and potentiostatic polarization tests. The electrochemical platform used is the AUTOLAB PGSTAT302N electrochemical workstation (Herisau, Switzerland), and the testing software is NOVA 2.0.

#### 2.3.3. Surface Hydrophobicity Assessment

The wettability characteristics of the bipolar plates, a critical factor influencing water management in fuel cells, were quantified using a contact angle analyzer (OSA200-B, Ningbo, China). Hydrophobic surfaces (θ > 90°) are preferred in bipolar plates to prevent electrolyte flooding and promote gas diffusion.

#### 2.3.4. Interfacial Contact Resistance (ICR) Test

**Figure 3:**
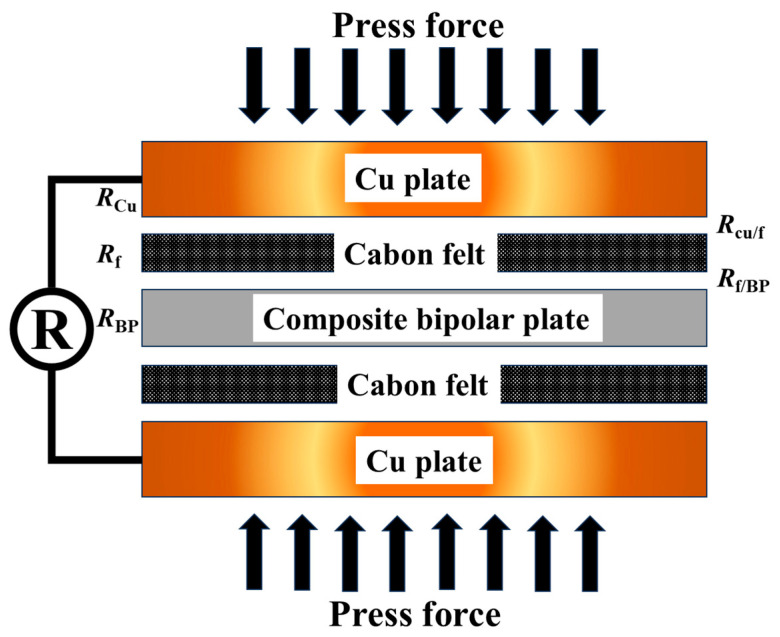
Graphic illustration of the assessment of bipolar plates’ conductivity.

The ICR between the bipolar plates and carbon paper was characterized using an improved Davies compression setup. The testing process is shown in Figure 3. The assembly comprised two highly polished copper current collectors (99.9% purity), sandwiching alternating layers of the composite sample and carbon paper (Tokyo, Japan). A servo-controlled hydraulic press applied compressive pressures ranging from 0.1 to 3 MPa, simulating actual fuel cell stack assembly conditions. A Keithley 2450 Source Meter recorded voltage (Solon, OH, USA) drops across the assembly at fixed current densities (50–200 mA·cm^−2^).

The connected voltmeter recorded resistance values as the pressure varied. The number of bipolar plates and carbon paper between the copper is specified as X and X + 1, respectively. The following formula can be used to determine the total resistance:

R_X_ = 2R_Cu_ + XR_BP_ + (X + 1) R_f_ + 2R_(Cu−f)_ + 2XR_(BP−f)_, (1)

In this case, R_X_ stands for the overall resistance, while $R_{f}$, ${XR}_{BP}$, and $R_{Cu}$ stand for the resistance of the carbon paper, bipolar plates, and copper, respectively. $R_{BP-f}$ represents the interfacial contact resistance (ICR) between the bipolar plates and the carbon paper, and it can be calculated using the following method: by increasing the number of copper plates by one, the increment in total resistance $R_{∆}$ can be calculated. The formula is as follows:

R_∆_ = R_(X+1)_ − R_X_ = R_BP_ + R_f_ + 2R_(BP−f)_, (2)

Here, the values of $R_{BP}$ and $R_{f}$ can be directly measured by a four-probe detector (RTS-9, Guangzhou, China).

Furthermore, the ICR can be determined using the following formula:

2R_(BP−f)_ = R_Δ_ − R_f_ − R_BP_, (3)

## 3. Results

### 3.1. Coating Characterization

**Figure 4:**
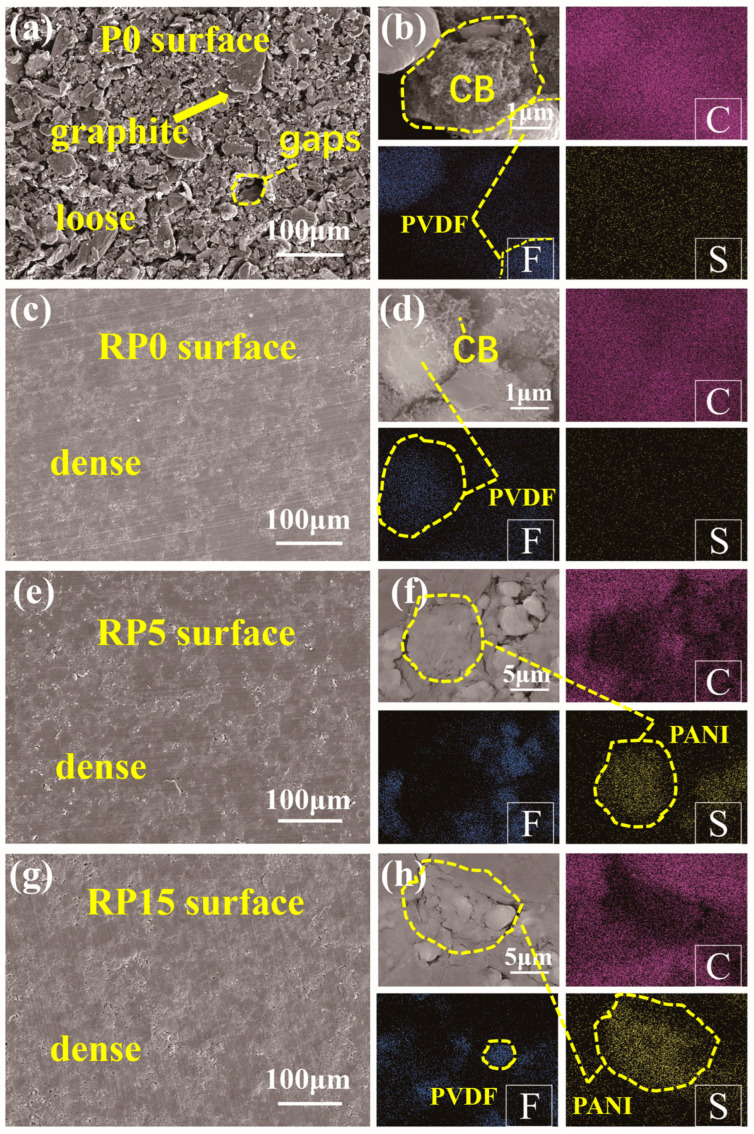
SEM images of the surface morphology of coatings: (**a**,**b**) P0, (**c**,**d**) RP0, (**e**,**f**) RP5, and (**g**,**h**) RP15.

Figure 4 presents the SEM microstructure images of four composite coatings. Additionally, EDS mapping was employed to observe the distribution of PVDF, CB, and PANI within the coatings. In Figure 4a, a lot of interlayer gaps were observed in the surface of the unrolled P0 sample with randomly arranged graphite flakes. EDS mapping of the C and F elements in Figure 4b shows the presence of aggregated PVDF particles.

In comparison, Figure 4c shows that the surface of the rolled RP0 coating is significantly dense. Under the influence of hot rolling, graphite flakes in RP0 were aligned in parallel, with reduced interlayer spacing and no visible gaps. PVDF resin is visibly distributed in the gaps between graphite layers, effectively filling the holes. Moreover, as shown by the C and F distribution in Figure 4d, the PVDF resin in the rolled coating is uniformly mixed with conductive carbon black, resulting in a homogeneous composite with indistinguishable boundaries between the two materials. Similar microstructural characteristics of the RP5 and RP15 coatings are illustrated in Figure 4e–h; however, PANI was observed as aggregating into flattened spherical particles (~5 μm in diameter) in these coatings. In contrast, the vast majority of PVDF particles maintain a spherical shape with a diameter of approximately 1 μm.

**Figure 5:**
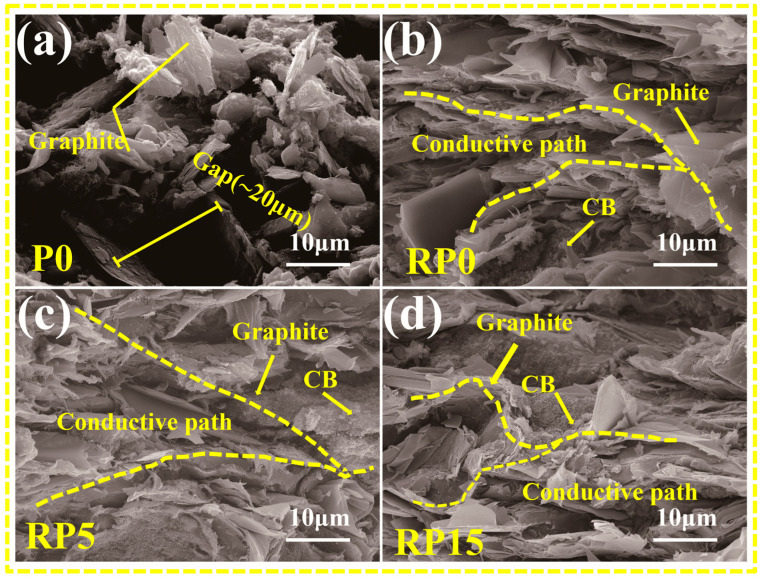
SEM images of the cross-sectional morphologies of the coatings: (**a**) P0, (**b**) RP0, (**c**) RP5, and (**d**) RP15.

Figure 5 presents the cross-sectional morphologies of the coatings. Prior to roll pressing, the graphite flakes in the P0 coating are randomly oriented, forming a loose and porous structure, as shown in Figure 5a. This leads to interlayer gaps of 20 μm between adjacent flakes. This loose arrangement impairs adhesion and conductivity, as electron transport primarily depends on graphite layers, with carbon black unable to bridge between layers effectively. Furthermore, large pores and disordered graphite layers offer minimal protection against corrosive agents, leading to poor corrosion resistance.

In contrast, Figure 5b–d reveals that the graphite layers in rolled coatings (RP0, RP5, and RP15) exhibit a distinct parallel orientation, with close interlayer contact and minimal porosity. PVDF filled with conductive carbon black occupies the gaps between graphite layers, further reducing the internal porosity and enhancing the barrier against corrosive agents. Additionally, carbon black connects adjacent graphite layers, forming a three-dimensional conductive network that enhances the coating’s overall conductivity.

### 3.2. Roughness

**Figure 6:**
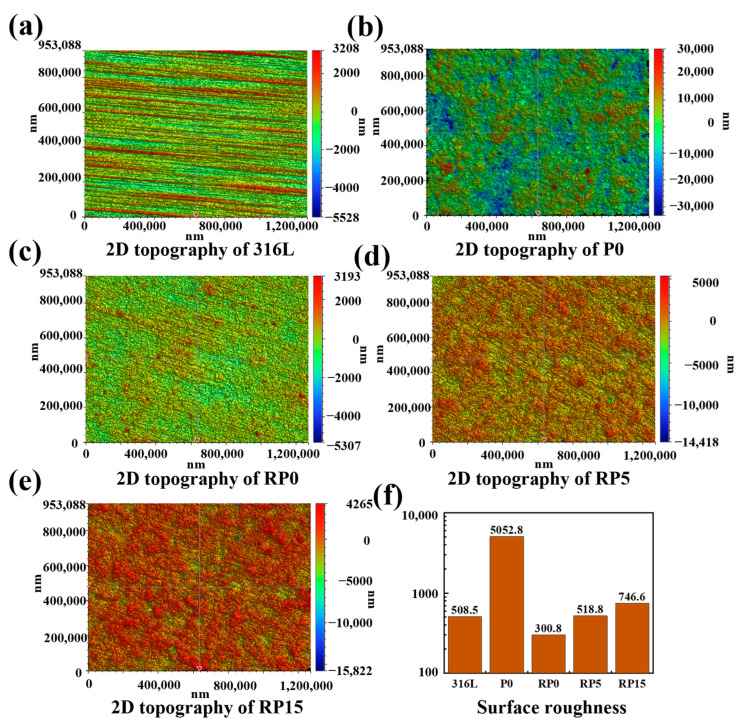
Surface topography of (**a**) 316L, (**b**) P0, (**c**) RP0, (**d**) RP5, (**e**) RP15, and (**f**) corresponding roughness data.

Surface roughness measurements of the samples were acquired via an optical profilometer, with conclusions shown in Figure 6. The surface roughness values for 316L, P0, RP0, RP5, and RP15 were 508.5 nm, 5052.8 nm, 300.8 nm, 518.8 nm, and 746.6 nm, respectively. The unrolled P0 coating was found to exhibit a significantly higher surface roughness compared to other samples, exceeding them by an order of magnitude. This increased roughness was attributed to the loose structure of graphite flakes on the coating surface, with flake size identified as the primary influencing factor.

The lowest surface roughness (300.8 nm) was observed in the RP0 sample, which shared the same composition as P0 but was processed by hot rolling. The surface roughness of the three rolled samples was similar to 316L SS, and was found to increase with higher PANI content. This trend is attributed to the significant reduction in surface roughness caused by the hot rolling process. Consistent with SEM observations, the graphite flakes in the rolled coatings were aligned parallel to the substrate surface, with PVDF tightly bonding the layers. Due to the thinness of the graphite flakes, the roughness of rolled samples was primarily determined by the size of the PVDF and PANI particles.

EDS analysis indicates that PANI tends to aggregate on the surface without melting during the hot rolling process. Consequently, larger PANI particles compared to PVDF were formed after rolling, explaining the increase in surface roughness with higher PANI content. Surface roughness is expected to impact the contact resistance and wettability of the samples [26,27,28].

### 3.3. Interfacial Contact Resistance (ICR)

**Figure 7:**
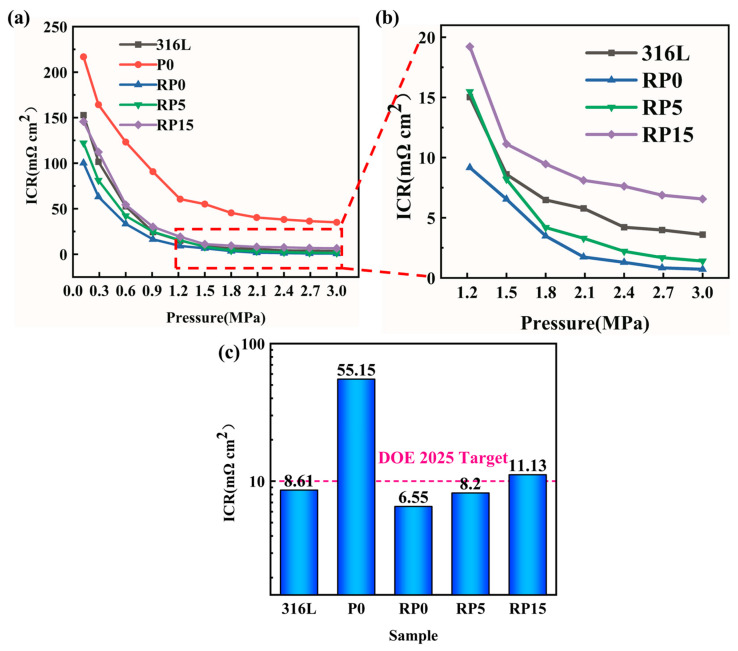
ICR values of 316L, P0, RP0, RP5, and RP15 samples, from 0.1 to 3.0 MPa (**a**) and (**b**) at 1.5 MPa (**c**).

The interfacial contact resistance (ICR) of the composite-coated plates was measured to evaluate the effects of the hot rolling process and PANI content on the conductive properties of the coatings. Figure 7a shows a sharp decrease in interfacial contact resistance (ICR) for all samples as the assembly pressure increases, followed by stabilization. However, the ICR values of the hot-rolled samples (RP0, RP5, RP15) were significantly lower than those of P0 across all pressures. In Figure 7, the ICR values at 1.5 MPa were measured as follows: bare 316L (8.61 mΩ·cm^2^), P0 (55.15 mΩ·cm^2^), RP0 (6.55 mΩ·cm^2^), RP5 (8.20 mΩ·cm^2^), and RP15 (11.13 mΩ·cm^2^). The highest ICR (55.15 mΩ·cm^2^) and poorest conductivity were recorded for P0, while the hot-rolled RP0 exhibited an 88.1% reduction in ICR (6.55 mΩ·cm^2^), demonstrating optimal conductivity. This improvement is attributed to the hot rolling process, which compacts the coating.

SEM analysis revealed that the graphite flakes in P0 were randomly oriented and loosely distributed, with large interlayer gaps and surface pores. In contrast, hot-rolled RP0 coatings displayed tightly packed, parallel-aligned graphite flakes (Figure 4c,d), forming a continuous conductive network. This ordered structure facilitates efficient electron transport along both in-plane and through-plane directions [29,30], establishing a three-dimensional conductive pathway that is critical for bipolar plates’ performance.

Meanwhile, a gradual increase in the ICR was observed with higher PANI content. The RP5 coating exhibited an interfacial contact resistance (ICR) of 8.20 mΩ·cm^2^, representing a 25.2% increase compared to RP0. This rise is linked to PANI’s lower intrinsic conductivity (~10 S/cm). Despite this rise, the ICR of RP5 remained lower than bare 316L SS, and it met the DOE 2025 target (<10 mΩ·cm^2^). In contrast, RP15 (11.13 mΩ·cm^2^) exceeded the DOE 2025 target by 11.3%, likely due to excessive PANI aggregation disrupting the conductive matrix.

### 3.4. Contact Angle

**Figure 8:**
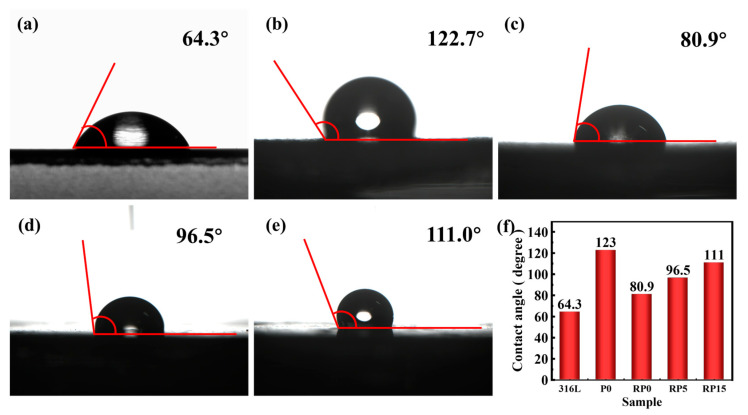
Contact angle of the samples: 316L (**a**), P0 (**b**), RP0 (**c**), RP5 (**d**), RP15 (**e**), and statistical chart (**f**).

The surface hydrophobicity of bipolar plates plays a critical role in both the corrosion resistance and water management efficiency in PEMFC. While increased hydrophobicity enhances the corrosion resistance, excessive levels may lead to liquid water accumulation and gas transport limitations [31]. In Figure 8, the bare 316L SS exhibits a contact angle of 64.3°, indicating a hydrophilic surface prone to water retention. This hydrophilic behavior may lead to channel flooding and elevate the corrosion risks in fuel cell stacks [32]. Meanwhile, the unpressed P0 sample, which contains no PANI, showed a significantly higher contact angle of 123°. This suggests that the P0 coating improved hydrophobicity, likely as a result of its lower surface energy and increased roughness. Moreover, roll pressing the same coating in the RP0 sample reduced the contact angle to 80.9°. This change is attributed to surface smoothing and decreased roughness from the pressing process, which limited hydrophobicity.

The addition of PANI in the RP5 and RP15 samples countered this decrease in hydrophobicity. For RP5 with moderate PANI content, the contact angle increased to 96.5°. This enhancement is attributed to the nanostructure of PANI, which increases surface roughness and reduces surface energy. In RP15, with a higher PANI content, the contact angle increased further, to 111°, indicating a highly hydrophobic surface. While high hydrophobicity improves corrosion resistance, excess amounts can lead to water accumulation in the form of large droplets, which may impede gas flow and degrade fuel cell performance [33]. Consequently, RP5 (96.5°) exhibited the optimal balance between water management and corrosion resistance. Its moderate PANI content, combined with a hot-rolled surface, resulted in optimal roughness and surface energy. This facilitated efficient water removal while preserving hydrophobicity and ensuring corrosion protection.

### 3.5. Electrochemical Measurement

**Figure 9:**
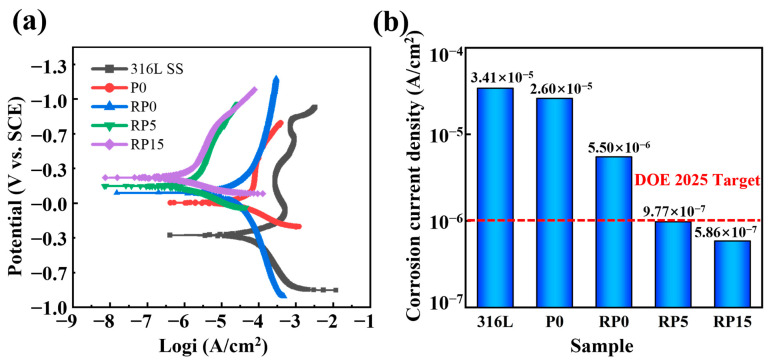
Potentiodynamic polarization curves of bare and coated 316L SS (**a**) and corrosion current density statistics of the samples (**b**).

Figure 9 shows the potentiodynamic polarization curves of the test samples, which are as follows: bare 316L SS, P0, RP0, RP5, and RP15. This method effectively evaluates corrosion resistance using the corrosion potential (*E*_corr_) and corrosion current density (*j*_corr_) derived from Tafel extrapolation. *E*_corr_, a fundamental thermodynamic parameter, assumes a pivotal role in the comprehensive evaluation of the corrosion-related properties of materials. It accurately mirrors the material’s vulnerability to corrosion. Under identical experimental conditions, a higher corrosion potential (*E*_corr_) indicates better corrosion resistance. In contrast, the corrosion current density (*j*_corr_) serves as a kinetic indicator, with lower values corresponding to slower corrosion rates and improved material performance [34]. The corrosion data derived from Tafel extrapolation for each sample are presented in the Table 2.

In Figure 9, the uncoated 316L SS displays the lowest *E*_corr_ and the highest *j*_corr_ among all samples, indicating poor corrosion resistance. In contrast, the *j*_corr_ values of coated 316L SS bipolar plates are significantly reduced, particularly for the hot-rolled samples (RP0, RP5, and RP15). Additionally, corrosion resistance improves progressively as the PANI content increases. Both RP5 and RP15 coatings meet the DOE 2025 target (<1 μA cm^−2^), with *j*_corr_ values of 0.977 μA cm^−2^ and 0.586 μA cm^−2^, respectively. These values represent a two-order magnitude reduction in the corrosion current density compared to bare 316L SS (34.1μA cm^−2^). Although RP15 exhibits superior corrosion resistance, its ICR exceeds the DOE 2025 target. In contrast, RP5 achieves both the ICR (<10 mΩ·cm^2^) and corrosion current density (<1 μA·cm^−2^) requirements, positioning it as a promising candidate material for bipolar plates in PEMFC.

**Figure 10:**
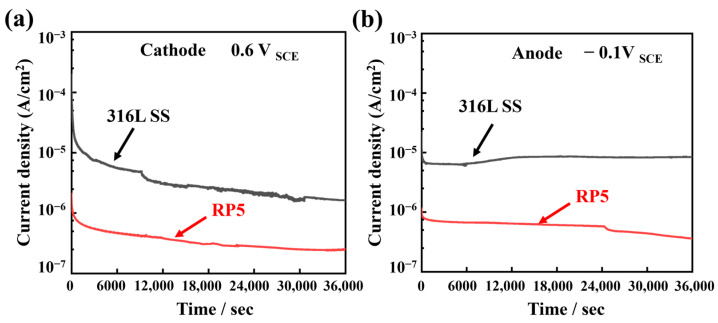
Potentiostatic polarization curves of 316L SS and RP5 in 0.5 M H_2_SO_4_ solution, (**a**) cathode (0.6 V_SCE_) and (**b**) anode (−0.1 V_SCE_).

To further investigate the corrosion resistance of RP5 under simulated PEMFC operating potentials, potentiostatic polarization tests were conducted on both RP5 and 316L SS for 10 h. The tests simulated cathode (0.6 V_SCE_) and anode (−0.1 V_SCE_) environments to replicate real-world fuel cell operating conditions. In Figure 10, the current density of both samples initially decreased rapidly and stabilized over time. Throughout the 10-h test, the RP5 coating exhibited consistently lower current densities than bare 316L SS. Under simulated cathode conditions, the current density of RP5 (0.25 μA·cm^−2^) was significantly lower than that of 316L SS (1.63 μA·cm^−2^). Similarly, under simulated anode conditions, the current density of RP5 (0.36 μA·cm^−2^) was markedly reduced compared to 316L SS (8.40 μA·cm^−2^). These results confirm that RP5 exhibits superior resistance to 316L SS in PEMFC operating environments, as evidenced by its sustained lower current density across both cathode and anode conditions.

**Figure 11:**
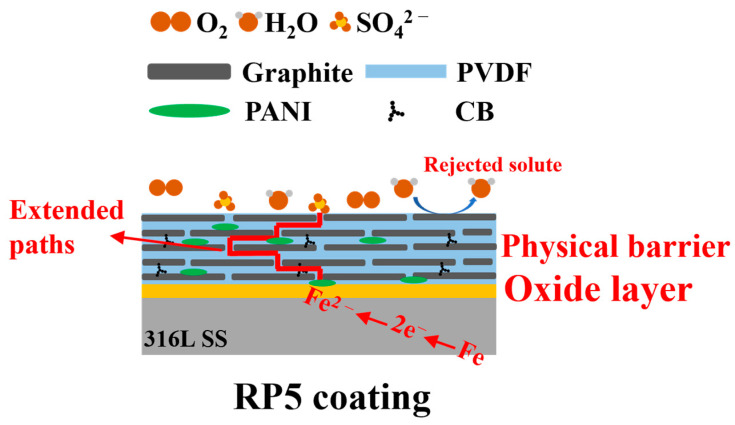
Schematic diagram of the corrosion protection mechanism of RP5 coating on 316L SS.

The enhanced corrosion resistance of RP5 is likely attributed to the synergistic interaction between its densely packed graphite layers and PANI. The schematic diagram of the corrosion protection mechanism of the RP5 coating on 316L SS is shown in Figure 11. Physically, the parallel-aligned graphite flakes act as a physical barrier, significantly extending the diffusion path of corrosive media [35]. Due to the large length-to-thickness ratio of the flakes, corrosive agents must navigate through narrow pores and follow a tortuous path along the flake surfaces before reaching the 316L substrate. This structure not only reduces the penetration rate of corrosive agents, but also establishes a robust protective mechanism. Additionally, the PANI in the coating is crucial for forming a protective oxide film on the 316L stainless steel surface. Through a reduction reaction, PANI supplies electrons, spontaneously and efficiently facilitating the oxidation of metal atoms to metal oxides under its redox-active influence. Moreover, PANI’s unique property enables it to maintain and rapidly repair this protective layer. When the oxide film is damaged in the dilute sulfuric acid environment, PANI quickly engages in redox reactions, re-oxidizing the exposed metal atoms to restore the film’s integrity. This self-repair mechanism ensures long-term protection and enhanced corrosion resistance [36].

## 4. Conclusions

A graphite/carbon black/PVDF composite coating with 5 wt% PANI (RP5) was successfully fabricated on 316L stainless steel via a scalable hot rolling process. SEM analysis confirmed the formation of a dense, defect-free coating on the substrate. Potentiodynamic polarization results revealed a corrosion current density of 0.977 μA·cm^−2^ for RP5, representing a two orders of magnitude reduction compared to 316L SS. Notably, during a 10-h potentiostatic polarization test, the RP5 coating maintained a consistently lower current density than 316L SS. Additionally, the RP5 coating achieved a low interfacial contact resistance (ICR) of 8.20 mΩ·cm^2^, fulfilling the DOE 2025 target. The results demonstrate that RP5 exhibits excellent comprehensive properties, positioning it as a promising candidate material for bipolar plates. Future studies should investigate the long-term corrosion behavior under simulated PEMFC operating conditions to validate its durability in practical applications.

## Figures and Tables

**Table 1:** Composition and preparation process of samples.

Sample	Graphite (wt%)	PVDF (wt%)	CB (wt%)	PANI (wt%)	Method
P0	65	30	5	0	blade
RP0	65	30	5	0	blade + hot rolling
RP5	65	30	5	5	blade + hot rolling
RP15	65	30	5	15	blade + hot rolling

**Table 2:** Self-corrosion voltage and self-corrosion current density of the sample.

Specimen	*E*_corr_ (mV)	*j*_corr_ (μA cm^−2^)
316L	−308	34.127
P0	4	26.074
RP0	99	5.498
RP5	165	0.977
RP15	245	0.586

## Data Availability

The original contributions presented in this study are included in the article. Further inquiries can be directed to the corresponding authors.
